# The Association Between Pre-Diabetes With Body Mass Index and Marital Status in an Iranian Urban Population

**DOI:** 10.5539/gjhs.v8n4p95

**Published:** 2015-07-30

**Authors:** Karamatollah Rahmanian, Mohammad Shojaei, Abdolreza Sotoodeh Jahromi, Abdolhossein Madani

**Affiliations:** 1 Research Center for Social Determinants of Health, Jahom University of Medical Sciences, Jahrom, Iran; 2 Research Center for Non-Communicable Diseases, Jahrom University of Medical Sciences, Jahrom, Iran; 3 Research Center for Social Determinants of Health promotion, Hormozgan University of Medical Sciences, Bandarabbas, Iran

**Keywords:** prediabetes, body mass index, education, marital status

## Abstract

Pre-diabetes increased the development of diabetes mellitus (type 2). The aim of study was to determine the association of body weight, education and marital status with pre-diabetes in an Iranian urban population. A sample of 788 subjects (360 men and 428 women) between the ages 30–85 years participated in our study and anthropometric measurements, educational level and fasting blood sugar of participants were recorded. The t and Chi square tests were used for continuous and categorical variables. The association of age, BMI categories, educational level and marital status to pre-diabetes was assessed by estimating the odds ratio. A p-value ≤ 0.05 were considered significant. The analysis was done using SPSS version 11.5. Our study showed that pre-diabetic subjects were older and low educated than normoglycemic subjects. Mean BMI and educational level were associated to pre-diabetes only in women. The odds of being pre-diabetes also were higher in obese women than in normal BMI women. No relationship was found between education and marital status with pre-diabetes in both men and women. Based on our finding, it is possible that advancing age and obesity has increased in pre-diabetes. This highlights the importance of population based survey to monitor blood glucose for effective prevention and control.

## 1. Introduction

Type 2 of DM (T2DM) is a most important public health crisis with a rising prevalence worldwide (Passa, 2002), because it is a common condition associated with increased morbidity and mortality (1998). The prevalence of T2DM is wide-ranging, from 1.2% to 14.6% in Asia (Azizi et al., 2003). A health survey that carried out during 2005 in Iran, used 89,400 subjects between 15-64 years old from the population of all province (Esteghamati et al., 2008), indicated that 7.7% of participants suffered from T2DM. In Iran, the prevalence of DM increased from 7.7% in 2005 to 8.7% in 2007 (Esteghamati et al. 2010). This rapidly-growing prevalence among developing countries is attributed to the urbanization (Al-Moosa et al., 2006; Shetty & Schmidhuber, 2006). Urbanization (Mbanya et al., 2010), prediabetes, older age and abdominal obesity (Kufe et al., n.d.), physical inactivity, positive family history of diabetes, hypertension and dyslipidemia increased the risk of diabetes (Nuhoğlu et al., 2015).

Persons with pre-diabetes have a 20-30% risk for growth of diabetes after 5-10 years (Meigs et al., 2003). In a study in Iran, the incidence of diabetes mellitus was 13.1% in persons with impaired fasting glucose (IGT) during 4 years (Hadaegh et al., 2005). Also, impaired fasting glucose or pre-diabetes increased the vrisk of cardiovascular disease (Levitzky et al., 2008).

According to result of studies, prediabetes associated with some factors. In some studies suggested that the obesity was associated with pre-diabetes (Hosler, 2009, Abtahi et al., 2010). In one study was shown that higher education was a risk of IGT only in women (Rathmann et al., 2005). Adversely, pre-diabetic subjects were low educated in compared to the control group (Javed et al., 2011). But other studies were found no significant association between education and IGT (Ko et al., 2001). Also in study was done in northeast of Iran by logistic regression analysis, reported that the IFG was not related to education and marital status (Azimi-Nezhad, Ghayour-Mobarhan et al., 2008).

Our study was done to assess the body weight, education and marital status relationship to pre-diabetic condition.

## 2. Materials and Methods

### 2.1 Study Area

In our cross sectional descriptive study, at the start, a multistage random sample of 892 subjects, aged ≥ 30 years was generated from Jahrom, Fars province at Southern Iran. Jahrom includes ten urban health centers that in each center, subjects were selected using the probabilities proportional to their region population.

From all participants was obtained signed informed consent and ethical approval was obtained from the Ethics Committee of the University of Jahrom. Pregnant women or participants with renal and hepatic disease were also excluded. Anthropometric measurement in accord with the standard procedures was measured by physician. Education was classified into high (university), medium (secondary or high school) and low (primary school or illiterate). Marital status was classified into three groups; married (the just way for couples to live together in Iran is by marriage), single (those who had never been married), and divorced and widowed.

### 2.2 Instruments and Measurements

#### 2.2.1 Anthropometry

Body weight was measured using Seca (Japan) to the near 0.1 kg with the participants wearing light clothing and without shoes. Height was measured to the adjacent 0.1centimeter using a portable stadiometer and with their heads in the Frankfort plane without shoes. Body Mass index (BMI) was calculated as weight divided by height squared (kg/m^2^).

#### 2.2.2 Blood Sugar

In early morning after an overnight fasting blood samples were obtained at the Peymanieh hospital and then assayed for serum sugar. A fasting blood sugar (FBS) < 100 mg/dl was considered as normal, values between 100 and <126 mg/dl were considered as prediabetes (or Impaired Fasting Glucose, IFG) in persons who were not on hypoglycemic medications (2003).

### 2.3 Statistical Analysis

One hundred and four persons were diabetic, thus 788 persons used for the analysis presented in this paper. For statistical analysis the BMI (kg/m^2^) was classified into three categories: normal weight <25, over-weight 25.0–29.9 and obese ≥30.0 kg/m^2^ (2000). To define the characteristics of subjects, descriptive statistics was used.

The t and Chi square tests were used for continuous and categorical variables. The association of age, BMI categories, educational level and marital status to pre-diabetes was assessed by estimating the odds ratio. A *p*-value ≤ 0.05 were considered significant. The analysis was done using SPSS version 11.5.

## 3. Results

Our study was showed that the pre-diabetic subjects were significantly low educated than persons with normal FBS (p= 0.013). There was no statistically significant difference in other variables except for age (p<0.001) between pre-diabetic and normoglycemic subjects (Table 1). Therefore, pre-diabetic subjects were about 7 years older than normoglycemic subjects.

**Table 1:** Descriptive characteristics of normoglycemic and pre-diabetic participants (n = 788)

	Normal FBS (n=648)		Pre-diabetes (n=140)	
	Continuous variables				
	Mean	SD	Mean	SD	p-value
Age (years)	47.8	13.6	54.7	13.6	<0.001
Body Mass Index (kg/m^2^)	26.1	4.4	26.6	4.3	0.25

	Categorical variables				
	Number	%	Number	%	

Sex					0.192
Male	289	44.6	71	50.7	
Female	359	55.4	69	49.3	
BMI categories					0.331
Normal weight	265	41	48	34.3	
Overweight	271	41.9	64	45.7	
Obese	111	17.1	28	20	
Marital status					0.211
Divorced or widowed	36	5.6	12	8.9	
Single	28	4.3	3	2.1	
Married	548	90.1	125	89.3	
Education					0.013
Low	256	39.5	79	50.0	
Medium	274	42.3	57	40.7	
High	118	18.2	13	9.3	

SD-Standard deviation.

Mean age was higher in pre-diabetic subjects than in subjects with normal FBS in both male and female (Table 2). There is a significant association of pre-diabetes with BMI groups only in female. In women, pre-diabetes was significantly associated with education. Therefore, the prevalence of low education was higher in pre-diabetic women than in women with normal FBS. Marital status and education were not association with pre-diabetes in men.

**Table 2:** Association of anthropometric parameters, education and marital status with pre-diabetes between male and female

variables	Male (n=360)					Female (n=428)				
	Normal		Pre-diabetes		p	Normal		Pre-diabetes		p
Continuous variables										
	Mean	SD	Mean	SD		Mean	SD	Mean	SD	
Age, year	50.1	13.9	55.6	13.5	0.002	46	11.9	53.7	13.7	<0.001
BMI (kg/m^2^)	24.8	4	24.9	3.7	0.858	27.2	4.5	28.3	4.2	0.049

Categorical variables										
	Number	%	Number	%		Number	%	Number	%	

BMI categories					0.156					0.100
Normal	152	52.8	32	45.1		113	31.5	16	23.2	
weight										
Overweight	108	37.5	35	49.3		163	45.4	29	42	
Obese	28	9.7	4	5.6		83	23.1	24	34.8	
Marital status					0.920					0.494
Divorced,	13	4.5	3	4.2	51	14.2	12	17.8
widowed or										
single										
Married	276	95.5	68	95.8		308	85.8	57	82.9	
Education					0.160					0.037
Low	113	39.1	36	50.7		143	39.8	34	49.3	
Medium	114	39.4	25	35.2		160	44.6	32	46.4	
High	62	21.5	10	14.1		56	15.6	3	4.3	

SD: Standard Deviation; yr: year.

In logistic regression analysis, the risk of pre-diabetes increased with advancing age in both women and men (Table 3). Obesity was significantly associated with increased risk of pre-diabetes only in women. No statistically significant association was found between pre-diabetes and marriage and education in both women and men.

**Table 3:** Determinants of pre-diabetes vs. normal fasting blood sugar from Binary logistic regression model

	Men			Women		
variables	OR	CI 95%	p	OR	CI 95%	p
Age, year	1.02	1.01-1.07	0.002	1.05	1.03-1.08	<0.001
BMI group, (normal wt, reference) Overweight Obese						
Overweight	1.68	0.97-2.92	0.065	1.30	0.66-2.59	0.442
Obese	0.78	0.25-2.41	0.668	2.34	1.13-4.85	0.022
Educational level (low, reference)						
Medium	1.54	0.68-3.46	0.297	3.23	0.93-11.15	0.064
High	1.61	0.70-3.70	0.261	1.76	0.47-6.50	0.394
Marital status	1.15	0.29-4.46	0.834	0.87	0.40-1.89	0.734

Variables entered on step 1: Age, marital status, Education, BMI groups. CI: Confidence Interval; OR: Odds Ratio.

## 4. Discussion

Pre-diabetes prevalence increased with raising BMI only in women. Also, Pre-diabetic prevalence inversely associated with educational level in both men and women. However with logistic regression, no relationship was found between education and marital status with pre-diabetes in both women and men.

In this study, the pre-diabetes was associated to BMI groups in the women. Similarly, Snodgrass et al suggested that fasting glucose was positively associated with BMI only in women (Snodgrass et al., 2010). Also, Bosi et al (Bosi, Carvalho et al. 2009) and Chin and Lin (Chen & Lin, 2010) reported the association between BMI and pre-diabetes. Other studies also found the positive relationship between BMI and pre-diabetes (Cao et al., 2010; Anjana et al., 2011). But, in study conducted by Lee et al (2011) and Gupta et al. (2008), there was no significant difference between pre-diabetes and BMI.

In our study pre-diabetes was not related to education in both sexes. Similarly, in previous studies among Hong Kong Chinese (Ko et al., 2001) and in Iran (Azimi-Nezhad et al., 2008), no significant relationship between education and impaired glucose tolerance (IGT) was found. Also, in another study, the education was not related to 2 hour glucose tolerance in subjects aged 35-74 years (Metcalf et al., 2008). Adversely, in the KORA survey 2000, among subjects aged 55-74 years, higher education was significantly associated with an increased risk of IGT in women (Rathmann et al., 2005). Also, in a study conducted in Australia, lower educated men had higher FBS and 2 hour glucose (Kavanagh et al., 2010).

In our study, the association of marital status with FBS or pre-diabetes was not found. In one study conducted in north of Iran, similar result was reported. In a Cohort study (Poljicanin et al., 2012), incident diabetes was related to being married (OR= 1.57, 95% CI: 1.08-2.28).

The present study may be limited by impending risk factors or confounders which were not accounted for in the analysis. Dietary and life style behavioral risk factors such as habitual physical activity could be some limitations that restricted our scientific contribution to the area.

Based on our finding, it is possible that advancing age and obesity has increased in pre-diabetes. This highlights the importance of population based survey to monitor blood glucose for effective prevention and control.

**Abbreviations**

BMI; Body mass index, FBS; Fasting blood sugar, IFG; Impaired fasting glucose, IGT; Impaired glucose tolerance.
